# A new type of aortic valved stent with good stability and no influence on coronary artery

**DOI:** 10.1186/1749-8090-8-210

**Published:** 2013-11-12

**Authors:** Jianzhi Cai, Haitao Huang, Yongxin Zhou, Yunqing Mei, Jie Shao, Yongwu Wang

**Affiliations:** 1 Department of Thoracic and Cardiovascular Surgery, Tongji Hospital of Tongji University, Xincun Road 389, Shanghai 200065, China; 2 Department of Thoracic and Cardiovascular Surgery, The Second Affiliated Hospital of Nantong university, Nantong, Jiangsu Province 226001, China

**Keywords:** Aortic valved stent, Transcatheter aortic valve implantation, Retrograde

## Abstract

**Background:**

To evaluated the feasibility and safety of new aortic valved stents in transcatheter aortic valve implantation (TAVI) using retrograde approach by *in vitro* testing and animal implantation.

**Materials and Methods:**

The fluid passing test, expanding and releasing tests, static and releasing tests in tube were performed for new valved stents. Transvalvular pressure gradient, effective orifice area, pre-implantation and post-implantation regurgitant volume for the new stents were detected. Then, the new stents were implanted in six pigs using retrograde approach. These pigs were euthanized 12 h after the implantation for anatomic evaluation.

**Results:**

*In vitro* tests showed that the closure of the new stents leaflets were effective, and stents could be released through catheter, then expanded completely and fixed fast in the tube. The coronary artery flow rates did not changed significantly after implantation (*P* > 0.05), while aortic regurgitant volumes were obviously reduced (*P* < 0.05). No significant difference in the transvalvular pressure gradient and effective orifice area of the new stents implanted within or above the valve leaflets was found (*P* > 0.05). *In vivo* experiments indicated that TAVI was successfully performed in six pigs using retrograde approach. However, one pig was died 10 h after the implantation since the stent was not expanded completely. The leaflets in stents were opening well and no valvular regurgitation was observed in the other five pigs. And thrombosis was not found.

**Discussion and Conclusion:**

The new type of aortic valved stent designed in this study was characterized with good stability and could avoid the impact caused by valve leaflets on the coronary artery.

## Background

Health care systems could be affected by cardiovascular disease (CVD) which is the main cause of death worldwide, accounting for 7.25 million deaths per year according to the report from the World Health Organization [1,2]. Meanwhile, diagnosis and treatment for diseases of the aortic valve are very essential since the aortic valve disease has high incidence and seriously threats the health and life of people. On the other hand, untreated diseases of aortic valve can ultimately lead to severe infection, heart failure, and even sudden death [3].

Currently, one of the most cardiac operations and standard therapies for aortic valve disease is open chest surgical aortic valve replacement which has mature procedures and good effects [4]. However, the surgical aortic valve replacement has some defects, such as severe trauma, extracorporeal circulation, long recovery time and long-term anticoagulation. A high prevalence of comorbidities is occurred in the elderly patients with symptomatic aortic valve stenosis [4]. What’s more, the common comorbidities including advanced age, previous cardiac surgery, left ventricular dysfunction, heart failure, pulmonary disease and renal insufficiency are known to be responsible for the high periprocedural and postprocedural risk of mortality and morbidity for patients [5,6]. And almost one third of patients with aortic valve stenosis who have these above comorbidities are rejected for surgery [7,8]. Therefore, it is still a great challenge for medical technicians to treat the aortic valve patients with high risk effectively.

The technology of transcatheter aortic valve implantation (TAVI) was first reported by Anderson et al. [9]. A new artificial aortic valve prosthesis which was prepared by mounting an expandable stent with a porcine aortic valve was implanted without thoracotomy or extracorporal circulation by the transluminal catheter technique indicating that transluminal catheter technique could be used for the implantation of artificial aortic valves in closed chest animals [9]. And Cribier et al. have reported that the first human implantation was performed in a man aged 57 with calcific aortic stenosis and many other associated noncardiac diseases [10]. Percutaneously implanted heart valve was successfully implanted within the native aortic valve with many diseases, with accurate positioning and a mild paravalvular aortic regurgitation, no impairment of the mitral valve function or the coronary artery blood flow was observed [10].

Though the success rate and curative effect for the TAVI is gradually increasing, the mortality rate of high-risk patients could still be up to approximately 20% within 30 days of the surgery [4,11,12]. However, TAVI has a series of advantages, such as minimally invasion, less physical damage and rapid postoperative recovery. Therefore, TAVI has good development and applied prospects especially in high-risk patients who have contraindications for surgical aortic valve replacement and it is increasingly being used to treat severe aortic stenosis in patients with high operative risk [13,14]. Despite the fact that TAVI has entered the mainstream as a viable treatment option for patients with symptomatic, severe aortic stenosis who are at prohibitively high surgical risk, there are still many problems associated with stent implantation including accurate and stable valve positioning, disorder of mitral valve function, risk of coronary artery obstruction and paravalvular leak [15-17]. Hence, the application of TAVI need to be further studied and developed.

In our study, a new type of aortic valve stent was made according to the anatomy parameters of porcine aortic root and porcine pericardium valves were sutured with the distal end of self-expandable stent. Furthermore, we evaluated the feasibility, safety and efficacy of the new type aortic valve stent in TAVI using retrograde implantation approach by *in vitro* testing and animal implantation studies.

## Methods

### Animal preparation

**Figure 1:**
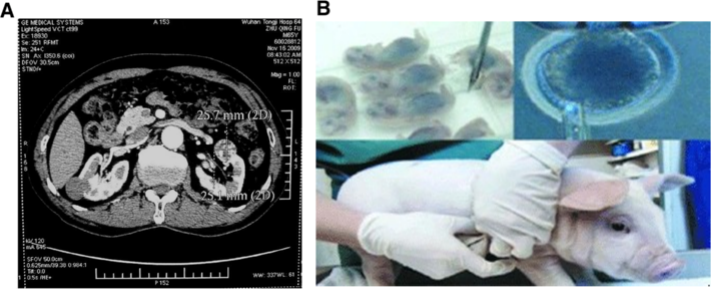
**The animal preparation for the implantation.** The healthy adult pig **(A)**; the aortic inner diameter of experimental pig **(B)**.

A total of six healthy adult pigs (Figure 1A) of either gender weighing 15–21 kg were enrolled (Songjiang Experimental Animal Field, Shanghai, China). The study was performed after the pigs were allowed to acclimate for 1 week in the Animal Experiment Center of Tongji University. Anesthesia for pigs was initiated by intramuscular injection of 10 mg/kg ketamine (Jiangsu Hengrui Medical Co. Ltd, China), followed by intravenous injection of 10 ml of 2.5% pentobarbital sodium (Sinopharm Chemical Reagent Co. Ltd, China). Animal care and use was in accordance with the guidelines established by the Animal Ethics Committee of Tongji University.

### Manufacture of the new type of aortic valved stent

**Figure 2:**
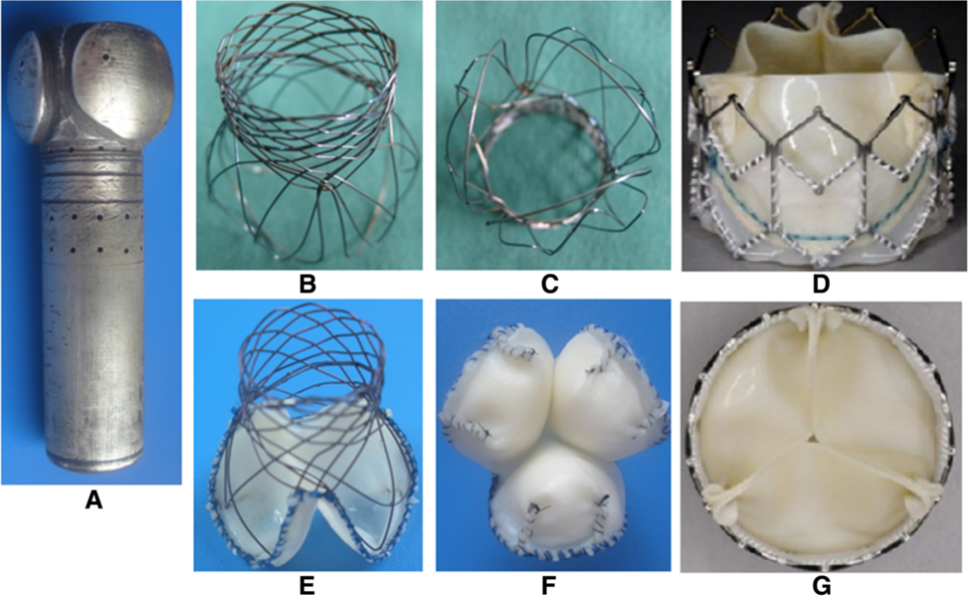
**Manufacture of the new type of aortic valved stent.** The module of aortic valved stent **(A)**; side view of the self- expandable stent **(B)**; top view of the self- expandable stent **(C)**; side view of the new aortic valved stent **(D)**; top view of the new aortic valved stent **(E)**. The old aortic valved stents in a side view **(F)** and a top view **(G)**.

The module of aortic valved stent (Figure 2A) was made according to the measured anatomy parameters and morphological characteristics of porcine aortic root (Figure 1B). The self-expandable stent with the diameter of 20–26 mm was made by 0.2 mm super malleable nitinol wire (Electric Titanium Factory of Jintai in Baoji City, Shaanxi, China) according to the method of sine curve. And the manufacture of self-expandable stent was accomplished (Figure 2B, C) after the procedures of heat treatment, heat setting (400-500°C, twice), acid pickling, cleaning, drying and inspection.

Fresh porcine pericardia (Songjiang Experimental Animal Field, Shanghai, China) with uniform thickness and fiber were selected and immersed in 0.6% glutaraldehyde for 36 h (pH = 7.4) after acellular disposal by 0.01% trypsin solution. Then, the decellularized pericardia were immersed in the 2% L-glutamic acid solution for 24 h to remove the toxicity of glutaraldehyde and sutured to the distal end of self-expandable stent by 6–0 PROLENE (Shanghai Pudong Jinhuan Medical Products Co. Ltd, China). The stents were placed in the fixing solution for one day after the sinuses of the valve were pressed with medical cotton balls. Then, the manufactured novel aortic valved stents (Figure 2D, E) were preserved in 70% ethanol and washed with physiological saline three times before use. Meanwhile, the old aortic valved stents with the cylindrical shape were also manufactured (Figure 2F, G).

### Performance of aortic valved stents in vitro

**Figure 3:**
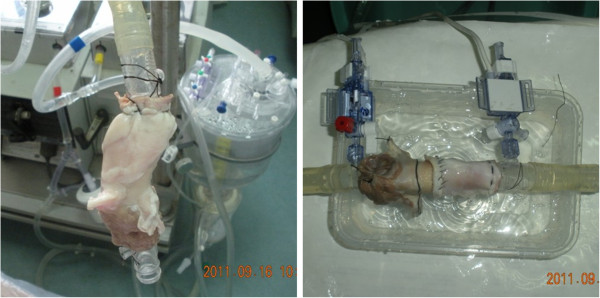
The static and releasing tests of aortic valved stent in PVC tube.

The valved stents were delivered to the left ventricle of the isolated heart from 3–4 months old miniature pig (Songjiang Experimental Animal Field, Shanghai, China) by ascending aorta and placed in the aortic valve. Then, the aortic valved stents were released and water was injected to observe the valvular competence and coronary flow rates (Figure 3).

The fluid passing test, expanding and releasing tests, static and releasing tests in tube were performed for new aortic valved stents by Sarns 5200 extracorporeal circulation at 40–120 mmHg (Sarns, America). In the fluid passing test, the closure, open and regurgitation of all valved stents were observed when uniform flow rushed to the stents in the opposite or same direction. In the expanding and releasing tests, the valved stents were released into the physiological saline at 37°C. Then, the expanding degree and rate were observed. For the static and releasing tests, the polyvinyl chloride (PVC) tube (Shanghai Xiangsheng Medical Instrument Co. Ltd, China) whose inner diameter was matched with that of the valved stent was selected. The stents were implanted in the tube by simulating TAVI at 37°C (thermostatic water tank, Sarns, America) via a catheter (Shanghai Pujiang Medical New Material Co. Ltd, China). The tube was filled with water continuously and stopped filling when the height of water surface was up to 150 cm. The fixation, flow direction, leakage, degree of closure and open of valved stents were observed. Transvalvular pressure gradient for the new type of valved stents (n = 6) implanted within the valve leaflets or in ascending aorta were measured under the flow rate of 1–9 L/min in the physiological saline at 37°C. Meanwhile, effective orifice areas for the new type of valved stents were also detected by the method of Doppler ultrasound. The pre-implantation and post-implantation regurgitant volume of new valved stents under 40–120 mmHg were detected. The static leakage and perivalvular leakage between the old and new valved stents under 40–120 mmHg were compared.

### Implantation of aortic valved stents in vivo

**Figure 4:**
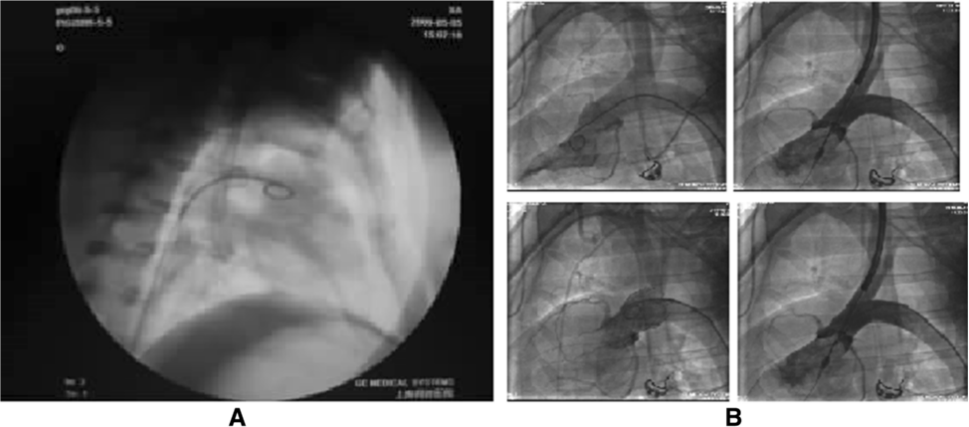
**Radiography of valved stent.** The radiographs of aortic valved stent in the ascending aorta **(A)** and during implantation **(B)**.

The experimental pigs were placed on the Model JL-1A operating table (Medical Equipment Factory of Shanghai Medical Instruments Co. Ltd, China) with supine position and hair was removed from the precordial region. After the skin of the inguinal region was sterilized, a 5 cm incision was made through the right inguinal skin and subcutaneous tissue. Then, the right femoral artery was subsequently exposed and punctured with a 7-French (F) leak-proof sheath. Heparin (100U/kg, Sinopharm Chemical Reagent Co. Ltd, China) was introduced via the sheath. A 6 F pigtail catheter was introduced through the right femoral artery sheath, and imaging of the left ventricle was performed by using digital subtraction angiography (DSA) apparatus (GE, America). The radiography of valved stent in the ascending aorta was shown in Figure 4.

Then, the positions of aortic valve and coronary artery were identified according to the imaging results. After the inner diameter of the ascending aorta was measured, the aortic valve was punctured. A balloon catheter (4.0 × 15 mm) was place in the crevasse of the aortic valve via a steel guide wire (2.5 m). The balloon catheter was pushed out after the crevasse of the aortic valve was enlarged by compressing the balloon dilator. The ascending aortic angiography was performed to confirm the aortic valve incompetence and echocardiography was conducted to measure the regurgitant volume.

New type aortic valved stents with the diameter of 2 mm larger than the measured ascending aorta were selected. The delivery route was established by introducing a steel guide wire into the left ventricle via the pigtail catheter. After the pigtail catheter was removed, a 12-16 F delivery sheath with an expandable stub was deployed into the ascending aorta at the top of the coronary artery via a guide wire. The expandable stub and steel guide wire were then removed and the 14 F sheath carrying the valve stent was connected with the delivery sheath. The stent was inserted into the delivery sheath positioned under the DSA apparatus to the previously marked position of the ascending aorta. After it was confirmed that the stent had been delivered to the optimal position, the delivery sheath was retracted to release the stent. The delivery sheath was subsequently removed and the right femoral artery was sutured.

Then, echocardiography and ascending aortic angiography were performed to observe aortic valvular regurgitation by deploying a pigtail catheter via the left femoral artery. The catheter and right femoral artery sheath were removed. All pigs received postoperative intramuscular injections of penicillin (80,000,000 U, North China Pharmaceutical Co. Ltd) and subcutaneous injections of heparin (2,500 IU).

### Statistical analysis

Statistical analysis was performed by the SPSS version 12.0 statistical software. The measurement data were expressed as mean ± standard deviation and analyzed by T-test. There is significantly statistical difference when *P* <0.05.

## Results

### Properties of aortic valved stents in vitro

*In vitro* performance tests, the closure of the valved stent leaflets were effective, and all fluid flows were not restricted in the opposite direction. The valved stents could be released through catheter, then expanded completely and could fix fast in the tube (Figure 3).

The coronary flow rates pre-implantation and post-implantation were shown in Table 1. The left coronary artery flow rate decreased from 25.38 ± 5.50 ml/s to 19.71 ± 4.80 ml/s (*P* = 0.0000) and the right decreased from 21.30 ± 5.41 ml/s to 17.74 ± 4.10 ml/s (*P* = 0.0001) when the new type of aortic valved stents were released within the native valve leaflets. The left coronary artery flow rate decreased from 27.71 ± 6.28 ml/s to 27.58 ± 6.16 ml/s (*P* = 0.07426) and the right decreased from 22.37 ± 4.85 ml/s to 21.73 ± 4.77 ml/s (*P* = 0.1487) with no significant difference when the new type of aortic valved stents were released in ascending aorta.

**Table 1:** The coronary flow rates pre-implantation and post-implantation

		**Pre-implantation (ml/s)**	**Post-implantation (ml/s)**	**Flow rate variation (ml/s)**	***P *** **value**
A	Left (n = 12)	25.38 ± 5.50	19.71 ± 4.80	5.67 ± 1.52	0.0000*
	Right (n = 12)	21.30 ± 5.41	17.74 ± 4.10	3.56 ± 1.88	0.0001*
B	Left (n = 12)	27.71 ± 6.28	27.58 ± 6.16	0.14 ± 1.35	0.7426
	Right (n = 12)	22.37 ± 4.85	21.73 ± 4.77	0.64 ± 1.36	0.1487

A: the new type of aortic valved stent released within the native valve leaflets; B: the new type of aortic valved stent released in the ascending aorta. The coronary flow rates pre-implantation *VS* post-implantation, *P < 0.05.

Furthermore, there was no significant difference in the transvalvular pressure gradient and effective orifice area of the new valved stents implanted within the native valve leaflets or in the ascending aorta (*P* > 0.05, Table 2). The transvalvular pressure gradients of the new valved stents implanted within the valve leaflets or in the ascending aorta were less than 1.33 Kpa (10 mmHg), and the effective orifice area was about 1 cm^2^ under the flow rate of 5 L/min (Table 2). Therefore, the opening of new valved stents could not be affected by valve leaflets. The post-implantation aortic regurgitant volumes under different pressures were obviously reduced (*P* < 0.05, Table 3). There was no significant difference in static leakage between the old and new valved stents under different pressures (*P* > 0.05, Table 4). The perivalvular leakages of new valved stents under 60, 80, and 100 mmHg were obviously lower than those of the old stents (*P* < 0.05, Table 4).

**Table 2:** Comparison of transvalvular pressure gradient and effective orifice area for the new type of valved stents implanted within the native valve leaflets or in the ascending aorta

**Flow rate (L/min)**	**Transvalvular pressure gradient (Kpa)**		**P value**	**Effective orifice area (cm**^**2**^**)**		**P value**
	**Within**	**Ascending aorta**		**Within**	**Ascending aorta**	
1	0.64 ± 0.09	0.63 ± 0.08	0.9087	0.77 ± 0.17	0.77 ± 0.15	0.8885
2	0.69 ± 0.10	0.70 ± 0.06	0.7785	0.82 ± 0.19	0.81 ± 0.16	0.8302
3	0.75 ± 0.09	0.76 ± 0.07	0.8057	0.86 ± 0.16	0.87 ± 0.19	0.4519
4	0.80 ± 0.08	0.78 ± 0.07	0.0643	0.92 ± 0.17	0.91 ± 0.16	0.8736
5	0.86 ± 0.08	0.83 ± 0.07	0.2431	1.00 ± 0.17	0.99 ± 0.17	0.6475
6	0.90 ± 0.09	0.91 ± 0.10	0.5549	1.03 ± 0.15	1.02 ± 0.15	0.7291
7	0.93 ± 0.10	0.96 ± 0.10	0.5165	1.09 ± 0.16	1.08 ± 0.16	0.9161
8	1.04 ± 0.09	1.06 ± 0.10	0.6707	1.13 ± 0.16	1.12 ± 0.17	0.5842
9	1.10 ± 0.09	1.12 ± 0.10	0.6892	1.13 ± 0.15	1.12 ± 0.16	0.5234

**Table 3:** Comparison of aortic regurgitant volume under different pressures between pre-implantation and post-implantation of new valved stents

**Pressure (mmHg)**	**Pre-implantation (ml)**	**Post-implantation (ml)**	**P value**
40	576.17 ± 51.71	514.83 ± 52.26	0.0193
60	703.50 ± 138.68	653.50 ± 117.14	0.0296
80	887.17 ± 150.84	791.50 ± 151.66	0.0422
100	985.34 ± 145.22	845.20 ± 132.31	0.0403
120	1243.07 ± 142.17	1095.36 ± 159.37	0.0395

**Table 4:** Comparison of the static leakage and perivalvular leakage for the old and new valved stents under different pressures

**Pressure (mmHg)**	**Valved stents (n = 6)**	**Total regurgitation (ml)**	**Static leakage (ml)**	**Perivalvular leakage (ml)**
40	Old	322.00 ± 57.30	151.50 ± 24.52	170.50 ± 41.28
	New	319.67 ± 59.61	135.83 ± 29.96	183.83 ± 47.62
	P value	0.9558	0.2943	0.6898
60	Old	511.50 ± 48.71	218.83 ± 32.82	292.67 ± 66.27
	New	476.17 ± 51.71	228.83 ± 38.97	247.33 ± 56.15
	P value	0.1190	0.6007	0.0220*
80	Old	700.17 ± 134.90	308.17 ± 84.37	392.00 ± 87.79
	New	654.50 ± 120.09	306.33 ± 93.18	331.50 ± 77.77
	P value	0.0195*	0.9205	0.0260*
100	Old	875.83 ± 170.19	358.17 ± 88.48	517.67 ± 117.83
	New	760.17 ± 129.37	346.50 ± 87.20	413.67 ± 100.39
	P value	0.0301*	0.2045	0.0373*
120	Old	1065.67 ± 149.71	399.17 ± 82.87	666.50 ± 114.87
	New	1035.00 ± 140.59	404.17 ± 83.56	630.83 ± 101.45
	P value	0.3376	0.7375	0.3969

*P < 0.05, new aortic valved stents compared with the old stents.

### Implantation of aortic valved stent *in vivo*

The implantation of aortic valved stent was successfully performed in six pigs using retrograde implantation approach and no obviously adverse reaction was observed after implantation. However, one pig was died 10 h after the implantation since the stent was not expanded completely. The leaflets in stents were opening well and no valvular regurgitation was observed after successful deployment of the stents in the other five pigs. Furthermore, there was no apparent change in the outflow tract pressure, systolic and diastolic function of left ventricle.

**Figure 5:**
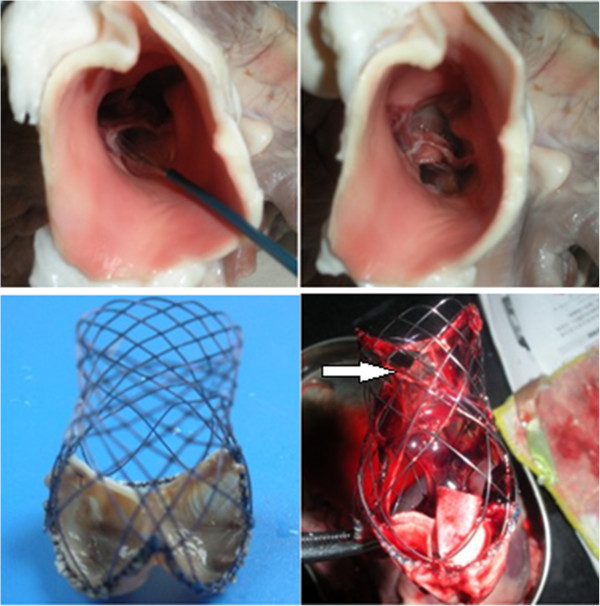
The post implantation morphological characteristics of aortic valved stent and the thin layer of endothelial cells on aortic valved stent was pointed out using white arrow.

**Figure 6:**
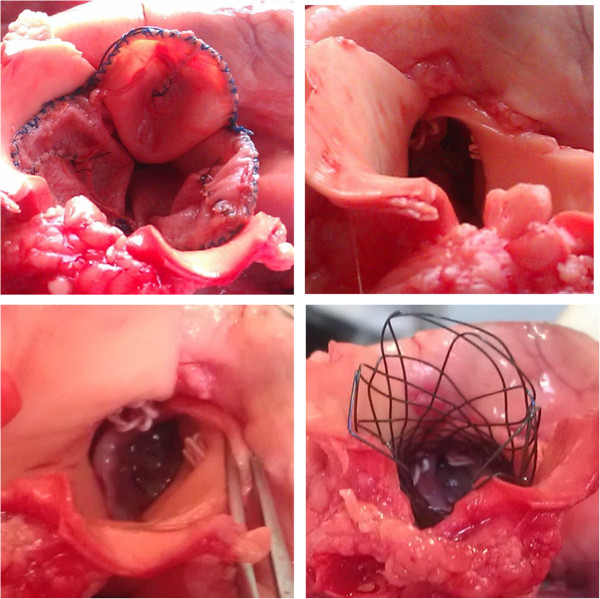
The post implantation anatomical features of three valve leaflets of aortic valved stent.

The other 5 pigs were euthanized 24 h post implantation for anatomic evaluation. The morphological characteristics of aortic valved stent were observed and most of the stent was covered with thin layer of endothelial cells (Figure 5). The post implantation anatomical features of three valve leaflets of aortic valved stent were shown in Figure 6. The aortic valved stents were fixed well in the ascending aorta. A small amount of fibroblast-like substances could observed in the aortic valved stents and thrombosis was not found.

## Discussion

TAVI was first proposed in 1992 by Andersen and Cribier reported the first clinical case of successful application of TAVI in 2002. Two typical representative aortic valved stents were Cribier-Edwards and CoreValve [18,19]. The Cribier-Edwards valve is a trileaflet valve composed of three equal sections of equine pericardium integrated into a stainless steel balloon expandable stent frame [20,21]. CoreValve is a self-expanding aortic valve prosthesis intended for retrograde delivery across the aortic valve [22,23]. It has been reported that the coronary artery flow could be effect by aortic valved stents when TAVI was performed in animal experiments and clinical treatments, especially in the cases of aortic insufficiency [24,25]. Flecher et al. have reported that implantation of a percutaneous valved stent in the orthotopic position with the native valve in place causes coronary ostial obstruction [26]. This problem highlights the need for modified stents that are designed for implantation in patients with non-retracted, fibrotic, or calcified leaflets.

In our study, we designed a new type of aortic valve stent according to the anatomy parameters of porcine aortic root and porcine pericardium valves were sutured with the distal end of self-expandable stent. The TAVI was performed in the isolated porcine heart *in vitro* and the coronary artery flow rates were measured. There was no significant difference in the left and right coronary artery flow rates between pre-implantation and post-implantation when the new type of aortic valved stents were released in ascending aorta (*P* = 0.7426, *P* = 0.1487). While, the left and right coronary artery flow rates decreased significantly after implantation when the new type of aortic valved stents were released within the native valve leaflets (*P* = 0.000, *P* = 0.0001). However, coronary ostial obstruction is mainly due to the calcification of the aortic valve leaflet since the calcific native valve leaflets are easily to be pressed toward the side wall by the stents [27,28]. Quaden et al. have reported that using a high-pressure water stream to endovascularly resect human calcified aortic valves could easily lead to thromboembolism since it is hardly to capture all the tissue fragments of disordered valves [29]. In our study, valved stents were tested in the healthy aortic valves. Therefore, whether the new type of aortic valve stent implanted in the ascending aorta could avoid the impacts caused by valve leaflets on the opening of coronary artery need to be further studied. No obvious shifting was observed when the new type of aortic valved stent was released within the native valve leaflets suggesting that the special shape of our aortic valved stent increased the stability at the aortic root. Therefore, a better stability of stent could be obtained when the aortic valve stent was implanted in ascending aorta.

TAVI is usually performed using the antegrade or retrograde implantation approach. Currently, the antegrade transseptal approach has been adopted in most cases. Cribier et al. have successfully performed the TAVI in patients with end-stage calcific aortic stenosis using the antegrade transseptal approach [10,27]. Boudjemline et al. performed retrograde replacement on the carotid artery of lamb which resulted in many complications including obstruction of coronary sinus, mitral insufficiency and stent shifting [30]. However, the efficacy of antegrade implantation approach for patients with severe heart diseases was not satisfied. Hanzel et al. reported that retrograde implantation was successfully conducted in a stable position for an 84-year-old man with critical aortic stenosis and refractory congestive heart failure, while many difficulties were encountered with an initial antegrade approach [31]. Webb et al. reported that the aortic valve area was significantly increased, no intraprocedural deaths, 16 patients (89%) remained alive after follow-up of 75 ± 55 days when TAVI was performed in 18 high risk patients (aged 81 ± 6 years) by using the retrograde implantation approach [32]. In our study, implantation of aortic valved stents *in vivo* further proved that the new type of aortic valved stent could be implanted in the ascending aorta by the retrograde implantation method via delivery catheter and successfully released. However, the stent did not expand completely in one pig. Hence, the development of shape memory functional stents which has relationship with the manufacture of the Ni-Ti alloy stent, suture of valves, size of stents and delivery catheter, need to be emphasized. What’s more, pigs were euthanized 24 h post implantation for anatomic evaluation. However, this design might be a limitation since only one time point was selected in our study. In our further study, we will design a serious of time points to follow-up the efficacy of the surgery.

## Conclusions

In conclusion, the new type of aortic valved stent designed in this study is characterized with good stability and has the potential to avoid the impact caused by valve leaflets on the coronary artery. In our study, the ascending aorta of the miniature pig was used as the animal model. However, the state of the valves in the isolated heart *in vitro* model was different from that in the heart of a live animal. And there must be some differences in the cardiac system between human and pigs though the cardiac structure of pig was similar to that of the human. Therefore, the application of aortic valved stent in human need to be further studied.

## Abbreviations

TAVI: Transcatheter aortic valve implantation; CVD: Cardiovascular disease; PVC: Polyvinyl chloride; DSA: Digital subtraction angiography.

## Competing interests

The authors declare that they have no competing interests.

## Authors’ contributions

JC and YW have made substantial contributions to conception and design, HH, YZ, YM, JS have made substantial contributions to acquisition of data, analysis and interpretation of data; JC have been involved in drafting the manuscript; YW revising it critically for important intellectual content and have given final approval of the version to be published. Each author should have participated sufficiently in the work to take public responsibility for appropriate portions of the content. All authors read and approved the final manuscript.
